# Hepatocyte Growth Factor Gene-Modified Adipose-Derived Mesenchymal Stem Cells Ameliorate Radiation Induced Liver Damage in a Rat Model

**DOI:** 10.1371/journal.pone.0114670

**Published:** 2014-12-12

**Authors:** Jiamin Zhang, Shiyuan Zhou, Yi Zhou, Feier Feng, Qianming Wang, Xiaolu Zhu, Huisheng Ai, Xiaojun Huang, Xiaohui Zhang

**Affiliations:** 1 Peking University People's Hospital, Peking University Institute of Hematology, Xicheng District, Beijing, China; 2 Department of Hematology, Affiliated Hospital to the Academy of Military Medicine Science, FengTai District, Beijing, China; CIMA. University of Navarra, Spain

## Abstract

Liver damage caused by radiotherapy is associated with a high mortality rate, but no established treatment exists. Adipose-derived mesenchymal stem cells (ADSCs) are capable of migration to injured tissue sites, where they aid in the repair of the damage. Hepatocyte growth factor (HGF) is critical for damage repair due to its anti-apoptotic, anti-fibrotic and cell regeneration-promoting effects. This study was performed to investigate the therapeutic effects of HGF-overexpressing ADSCs on radiation-induced liver damage (RILD). ADSCs were infected with a lentivirus encoding HGF and HGF-shRNA. Sprague-Dawley (SD) rats received 60Gy of irradiation to induce liver injury and were immediately given either saline, ADSCs, ADSCs + HGF or ADSCs + shHGF. Two days after irradiation, a significant reduction in apoptosis was observed in the HGF-overexpressing ADSC group compared with the RILD group, as assessed by terminal deoxynucleotidyl transferase dUTP nick end labeling (TUNEL) staining. Scanning electron microscopy showed chromatin condensation after irradiation, which was ameliorated in the group that received ADSCs and was reversed in the group that received HGF-overexpressing ADSCs. HGF-overexpressing ADSCs ameliorated radiation- induced liver fibrosis through down regulation of α-SMA and fibronectin. Hepatocyte regeneration was significantly improved in rats treated with ADSCs compared with rats from the RILD group), as assessed by Ki-67 immunohistochemistry. Rats that received HGF-overexpressing ADSCs showed an even greater level of hepatocyte regeneration. HGF-overexpressing ADSCs completely blocked the radiation-induced increase in the enzymes ALT and AST. The effect of mitigating RILD was compromised in the ADSC + shHGF group compared with the ADSC group. Altogether, these results suggest that HGF-overexpressing ADSCs can significantly improve RILD in a rat model, which may serve as a valuable therapeutic alternative.

## Introduction

Radiotherapy is one of the major effective treatments for primary or metastatic liver cancers. However, normal liver tissues, especially those in active metabolic and regeneration states, will experience collateral damage, which poses a vital limitation to the application of radiotherapy [1]. Irradiation can result in distinct metabolic alterations in hepatic functions and induce the carbonylation of specific liver enzymes. The oxidation of liver enzymes may underlie some radiation-induced alterations in hepatic function [2]. In addition to impaired liver function, increased apoptotic cell proportion, decreased hepatocyte number and fibrosis were also observed in irradiated livers [3]. Approximately 5 to 10 percent of the patients who receive a radiation dose in excess of 30Gy develop radiation-induced liver damage (RILD) [4], [5], [6]; when the dose is increased to 43Gy, the prevalence of RILD increases to 50% [7]. Currently, treatment for RILD is not well established. Although the use of anticoagulants and steroids has been suggested, and although supportive treatments seem to have shown some positive efficacy, a substantial fraction of the patients will eventually die from liver failure [8].

A large number of studies have drawn attention to mesenchymal stem cells (MSCs) due to their potential for tissue repair in a wide range of tissue types. Moreover, MSCs specifically migrate to radiation-injured tissues due to the activation of molecular pathways that up regulate the expression of chemokines [9]. Therefore, MSC therapy may be a promising therapeutic approach to improve radiotherapy-induced tissue injury. The mechanisms of MSC radioprotections against liver damage consist of trophic effects, anti-oxidative and vasculature protection [10]. Among various mesenchymal stem cell lines, adipose-derived stem cells (ADSCs) appear to be a preferable source for cell-mediated therapy. ADSCs are highly self-renewing multipotent mesenchymal cells [11] that are abundantly available and can be easily harvested [12]. Furthermore, ADSCs have been proven to have a profound impact on the improvement of liver injuries [13], [14], [15] as well as on other types of tissue injury [16], [17]; therefore, ADSCs are one of the best candidate cell lines to use as vector cells to rescue liver tissue injured by irradiation.

ADSCs can secrete many growth factors, such as hepatocyte growth factor (HGF), and contribute to tissue remodeling through paracrine mechanisms rather than by cellular differentiation [18], [19]. Of these growth factors, HGF is a multifunctional tissue growth factor and a vital cytokine for the promotion of hepatocyte regeneration [20]. HGF also plays an indispensable role in the prevention of tissue fibrosis and apoptosis [21], [22], [23]. In fact, apoptosis and tissue fibrosis are among the pathologic consequences that can result from irradiation. Zhu et al. reported that ADSCs that overexpress HGF exerted a better therapeutic effect in a rat model of acute myocardial infarction [24]. Cai et al. [25] suggested that the efficacy of ADSCs in the repair of ischemic tissue is compromised by the down regulation of HGF expression; they demonstrated that paracrine support accounts for a substantial portion of the in vivo benefit produced by ADSCs. This evidence suggested to us that an elevation in the expression of HGF may enhance the therapeutic potential of ADSCs. Similarly, the mesenchymal stem cells from human umbilical cord blood over-expressing hepatocyte growth factor prevent liver damages in rats [26]. In addition, lentiviral vectors, which outperform traditional therapeutic vectors in their endurance and efficiency, could introduce permanent and high expression of HGF in ADSCs.

Given the implications of this research, we hypothesized that ADSCs that overexpress HGF can inhibit apoptosis of hepatocytes, reverse liver fibrosis and promote the regeneration of hepatocytes in a rat model of RILD. The mechanism involved may be modulated through the secretion of HGF due to its multiple biological functions. Therefore, in the current study, we assessed whether ADSCs that overexpress HGF could alleviate liver damage induced by irradiation. Furthermore, we examined whether this amelioration was mediated through HGF expression.

## Materials and Methods

### Isolation and Culture of Adipose-Derived Stem Cells

All procedures were approved by the animal care committee of People's Hospital, Peking University (Permit Number: 201301). Sprague-Dawley rats weighing approximately 180 to 200 g were euthanized using CO_2_ upon termination of the study. The procedures were performed as previously reported with some modifications [27], [28]. Briefly, aseptically harvested rat adipose tissue was extensively washed with phosphate-buffered saline, finely minced, and digested for 60 min at 37°C with type I collagenase (Sigma, St. Louis, USA). The cell suspension was then neutralized with Dulbecco's Modified Eagle's medium (DMEM, Gibco, USA) containing 10% fetal bovine serum (Gibco, New York, USA), and 100 U/mL penicillin/streptomycin. After filtration through a 70-µm filter and centrifugation at 1500 rpm for 5 min, the pellet was resuspended. The cell suspension was plated in 75-cm^2^ cell culture flasks (Corning, New York, USA) and maintained at 37°C in a humidified atmosphere of 5% CO2; the medium was changed every 2 days. ADSCs were passaged at 80% confluence with 0.25% trypsin at 37°C, and all cells were used for analysis at passages three to five.

### ADSC Surface Antigens Measured by Flow Cytometry

Cells at passage three were digested, washed with PBS and incubated with antibodies against rat CD31-PE (BD Biosciences, New Jersey, USA), CD34-PE (Santa Cruz, Dallas, USA), CD45-FITC (BD Biosciences, New Jersey, USA), and CD90-PE (BD Biosciences, New Jersey, USA), as well as with isotype IgG1-PE (BD Biosciences, New Jersey, USA) and isotype IgG1-FITC (BD Biosciences, New Jersey, USA) for 30 min. Cells were then washed and centrifuged at 1500 rpm for 5 min. Finally, cells (5×10^5^/ml) were suspended in PBS and analyzed by flow cytometry.

### Construction of Recombinant Lentiviral Vectors

The plasmid encoding human HGF or shHGF was purchased from OriGene (OriGene, Rockville, USA). The lentiviral vector system (GeneChem, Shanghai, China) was composed of three parts for viral packaging, including the pGC-E1 vector, the pHelper 1.0 vector and the pHelper 2.0 vector. The full-length cDNA of hHGF was cloned into the pGC-E1 vector by digestion with AgeI and ligation of the resultant fragments into the AgeI site of the pGC-E1 vector (pGC-E1-hHGF). PCR and DNA sequencing confirmed the accurate insertion of hHGF cDNA. pHelper 1.0 plasmid DNA (15 µg), pHelper 2.0 plasmid DNA (10 µg), and pGC-E1-hHGF, pGC-E1-shHGF or PGC-E1-Fluc plasmid DNA (20 µg) were co-transfected into subconfluent 293T cells using Lipofectamine 2000 (Invitrogen, Carlsbad CA, USA). Infectious lentiviruses were harvested 48 h post-transfection. Viral vector titers of recombinant lenti-hHGF and lenti-shHGFwere measured by fluorescence-activated cell sorting analysis of the 293T cells. The viral titer was 10^8^ transducing units/ml of medium.

### Infection of ADSCs with the Lentiviral Vectors

At the third passage, the cell medium was removed prior to the infection, and then, ADSCs were incubated with lenti-hHGF, lenti-shHGF or lenti-GFP in special polybrene-containing (5 µg/ml) growth medium. On the day of infection, cells were plated along with lenti-HGF, lenti-shHGF or lenti-GFP at different multiplicity of Infection (MOI) in serum-free growth medium containing 5 µg/ml polybrene. Serum-containing growth medium was added after 4 h and then replaced after 48 h. Subsequently, 4–5 days post-infection, reporter gene expression was examined using fluorescence microscopy. ADSCs were plated in T-75 flasks and infected at an MOI of 100 for transplantation. Finally, ADSC hHGF and ADSC shHGF cells were passaged and prepared for cell transplantation.

### Establishment of a Rat Model of Radiation-Induced Liver Damage

All animals were treated according to protocols approved by the animal care committee of People's Hospital, Peking University (Permit Number: 201301). Sixty 6-week-old, male Sprague-Dawley rats weighing approximately 180 to 200 g were intraperitoneally anesthetized with 3 ml of 10% chloral hydrate per kilogram of body weight. The hepatic region of each rat was determined using an ultrasonic detector and was marked on the skin. Rats received whole-liver irradiation with a single dose of 60Gy (dose rate of 215.22 cGy/min) using a HFY-YC Co-60 γirradiator (REVISS, Buckinghamshire, UK). The other parts of the body were protected from the irradiation by lead shielding [29]. Animals were randomly divided into four groups. Group 1 (n = 15) consisted of rats that received 60Gy of whole liver irradiation and was defined as the RILD group. The remaining three groups (n = 15) were given multiple caudal vein injections of ADSCs, ADSCs infected with lenti-HGF or ADSCs infected with lenti-shHGF (1×10^7^ cells in 0.3 ml PBS for each rat) immediately after irradiation and were defined as the ADSC group, the ADSC + lenti-hHGF group and the ADSC + lenti-shHGF group, respectively. Another group of ten rats without irradiation or therapy served as controls.

### Detection of Apoptosis by Transmission Electron Microscopy

The liver samples were fixed in 3% glutaraldehyde for 6–8 h at 4°C. The samples were post-fixed in 0.1 M phosphate buffer containing 1% osmium tetroxide for 2 h at 4°C, dehydrated in ascending grades of acetone, infiltrated and embedded in Araldite CY212 and polymerized at 60°C for 72 h. Thin (60–70 nm) sections were cut with an ultra-microtome. The sections were mounted on copper grids and stained with uranylacetate and lead citrate and were observed using a Morgagni 268 D transmission electron microscope (FEI Company, Portland Oregon, USA).

### Detection of Apoptosis by TUNEL Staining

Liver specimens were paraffin-embedded after fixation in paraformaldehyde for 24 h. Tissues were collected, cryopreserved, and sectioned for histologic staining. Radiation-induced cell apoptosis was assessed by TUNEL staining using an in situ cell death detection kit (Roche Applied Science, Indianapolis IN, USA) according to the manufacturer's protocol. Briefly, tissue sections were fixed in 4% paraformaldehyde for 10 min followed by washing in PBS and blocking with 3% H_2_O_2_ in methanol for 10 min. After permeabilization with a solution containing 0.1% Triton X-100 and 0.1% sodium citrate, sections were labeled with 25 µL of TUNEL reaction mixture containing a 1∶2 dilution of enzyme for 2 h at 37°C in a humidified chamber. Signals were then converted into horseradish peroxidase and were visualized. Five randomly selected areas were chosen during the microscopic examination for comparisons between each experimental group.

### Histologic Liver Evaluation

The hepatic specimens were fixed in 4% paraformaldehyde and embedded in paraffin. Liver sections were stained with hematoxylin-eosin or were subjected to Masson's trichrome stain and immunohistochemistry with antibodies against GFP (Abcam, Cambridge, UK), fibronectin (Abcam, Cambridge, UK), α-SMA (Santa Cruz, Dallas, USA), CK-7 (Abcam, Cambridge, UK), CK-19 (Proteintech, Chicago, USA) and Ki-67 (Abcam, Cambridge, UK).

### Real-Time Quantitative Reverse Transcriptase-Polymerase Chain Reaction (RT-PCR) Analysis

To measure the levels of HGF mRNA, total RNA (50 ng) was isolated from cells using TRIzol reagent (Invitrogen, Carlsbad CA, USA). Real-time RT-PCR was performed using a QuantiTect SYBR Green RT-PCR kit (QiagenInc, Valencia CA, USA), and specific primers were chosen according to the manufacturer's suggested conditions. The levels of mRNA were normalized to control.

### ELISA

Four days after transfection, the supernatants of multiple transfected ADSCs were collected and centrifuged at 1500 rpm for 5 min at +4°C and then stored at −80°C. Liver tissue weighing approximately 100 mg was rapidly excised immediately after the animals were killed and rinsed in PBS. After homogenization with a glass rod, the tissues were centrifuged at 4000 rpm for 10 min at +4°C. The supernatants were collected and stored at −80°C. The amounts of HGF in the tissue supernatants and cell supernatants were measured using a commercial ELISA kit (R&D, Minneapolis MN, USA) according to the manufacturer's instructions.

### Serum Liver Enzymes

Blood samples were collected 2 days post-irradiation. Serum alanine aminotransferase (ALT) and aspartate aminotransferase (AST) activities were assessed using a commercial kit (BioVision, Milpitas, USA). Ten microliters of each serum sample were used according to the manufacturer's protocol.

### Western Blot

A western blot was used to measure the protein expression of collagen-I. The total protein was extracted from the liver specimen with lysis buffer and protease inhibitor cocktails; then, SDS and PAGE gels of the cell lysates were probed with collagen-I antibodies (Santa Cruz, Dallas, USA). The detection was performed using enhanced chemiluminescence. A quantitative analysis was performed by scanning the blots and calculating the relative intensities in relation to the corresponding actin signal using Quantity One (Bio-Rad, Hercules CA, USA).

### Statistical Analysis

SPSS Statistic software (IBM Corporation, New York, USA) was used for the general statistical analysis. All data were presented as the mean ± SD. Significant differences in the numerical data between the groups were determined by one-way ANOVA, after which a comparison between the groups was performed using the LSD test. Values of p<0.05 were considered statistically significant.

## Results

### Construction of HGF Gene-Modified ADSCs

**Figure 1:**
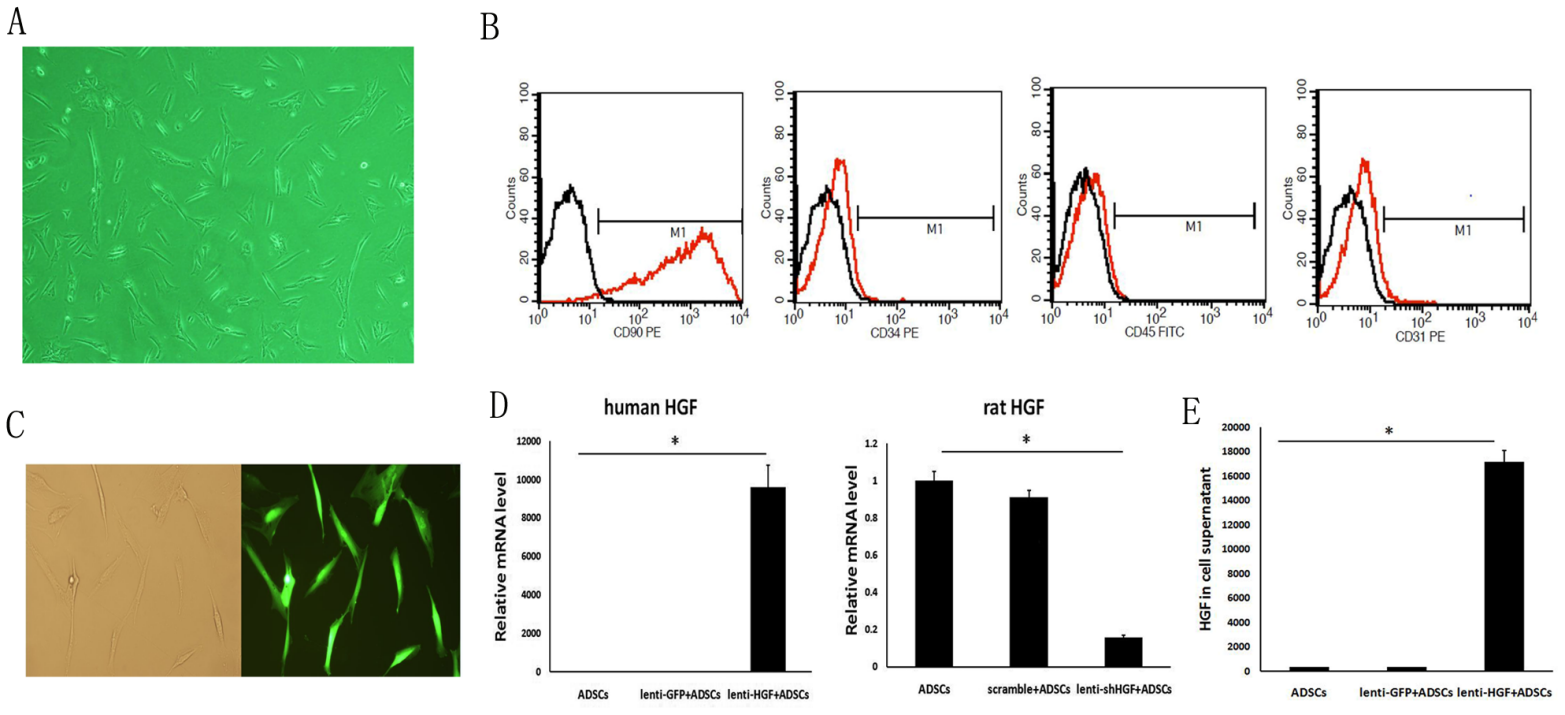
Construction of HGF gene-modified ADSCs. (A) Micrographs of cultured ADSCs at passage three. (B) Surface antigens of ADSCs were analyzed by flow cytometry. Freshly cultured ADSCs were positive for CD90 (99%), but not for CD31 (0.02%), CD34 (0.02%), or CD45 (0.03%). (C) Infection efficiency 4 days post-infection. GFP-expressing ADSCs were detected and counted using fluorescence microscopy. The infection efficiency was 100%. (D) Expression of HGF mRNA as detected by RT-RCR 4days post-transfection. (E) Expression of HGF protein as detected by ELISA 4days post-transfection. *p<0.05.

After 4–5 hours in culture, a few dispersed cells were observed to be adherent on the bottom of the dish (Fig. 1A). Cells began to grow quickly after 3days and developed long spindly processes, occupied a small volume, and did not grow in any particular direction. The observations of cell morphology under a light microscope fit the characteristics of ADSCs. After the third passage, FACS analysis was used to verify the phenotype. The results showed that our cultured cells were positive for CD90 (99%) but were negative for expression of CD34 (0.02%), CD45 (0.03%) and CD31 (0.02%) (Fig. 1B).

ADSCs were divided into four groups at passage three and were transfected with lentivirus carrying HGF-GFP, shHGF-GFP or GFP alone. We used fluorescence microscopy to observe expression of the reporter gene. GFP-expressing ADSCs were observed under a microscope (Fig. 1C); the transfection efficiency was 100%.

To further verify that the lentivirus was successfully transfected into ADSCs, RT-PCR was used to detect mRNA levels 4 days after transfection. The results demonstrated that the expression of HGF in the lenti-HGF group was clearly higher than that in the lenti-GFP group (9588.60±1189.53 vs. 1.00±0.32, p<0.05) (Fig. 1D); in the lenti-shHGF group, the expression of HGF, consistent with our expectations, was distinctly lower than that of the control group (0.16±0.01 vs. 1.00±0.05, p<0.05) (Fig. 1D). ELISA demonstrated that the expression of HGF in the cell culture supernatant in the lenti-HGF group was obviously higher than that in the lenti-GFP group (17181.8±900.4 vs. 372.2±11.8 pg/ml, p<0.05) (Fig. 1E).

### HGF Gene-Modified ADSCs Migrate to Damaged Tissue Sites

**Figure 2:**
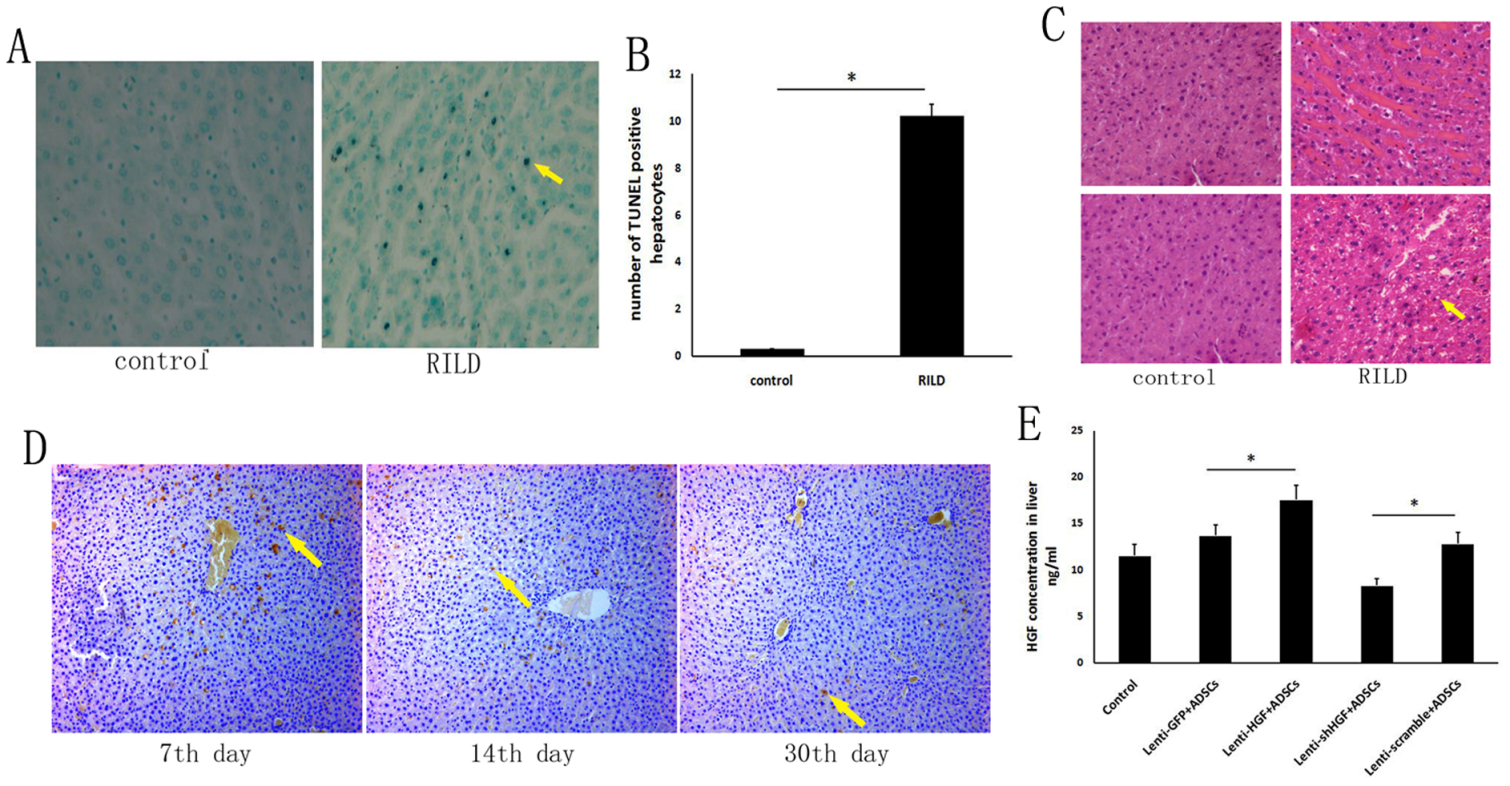
HGF gene-modified ADSCs migrate to damaged tissue sites. (A) TUNEL staining of liver sections from the control group and liver sections from the RILD group 2 days post-irradiation. Arrows indicate hepatocytes that are positive for TUNEL staining. (B) Quantification of TUNEL-positive cells. Five fields were randomly selected from each specimen, and the number of TUNEL-positive cells was determined (×400). (C) HE stain of liver specimens obtained from the control group and from the RILD group 60 days after irradiation. Arrow indicates vacuolar degeneration in liver. (D) ADSCs expressing GFP were detected in rat liver 7, 14 and 30 days after transplantation (100×). Arrows indicate cells positive for GFP staining. (E) HGF was detected by ELISA in rat liver 2 weeks after transplantation. *p<0.05.

TUNEL staining revealed an increased level of apoptosis in the RILD group (Fig. 2A). The number of TUNEL-positive hepatocytes in the RILD group was significantly higher than that of the control group (3.7±0.8 vs. 1.5±0.2, *p*<0.05) (Fig. 2B). Electron microscope images showed changes typical of cellular apoptosis in tissues from the RILD group 2 days post-irradiation. Liver fibrosis was observed 8 weeks after 60 Gy-irradiation by HE-staining of tissues from the RILD group, which was not observed at that time in the control group (Fig. 2C). A biopsy showed shrunken livers with irregular edges in rats from the RILD group. Furthermore, we tagged the GFP marker to trace the in vivo distribution of ADSCs after transplantation. By the 7th day post-transplantation, the majority of the GFP-positive cells were concentrated around the portal region. The mean density of the GFP-positive cells was 37.33±33.13/mm^2^ by day 7. However, by day 15, the GFP-positive cells began to migrate into the hepatic lobule region. The density of the GFP-positive cells was 23.33±19.96/mm^2^ by day 15. By day 30, the GFP-positive cells could still be observed in both the portal and lobule regions. The density of the GFP-positive cells was 6.50±5.06/mm^2^ by day 30 (Fig. 2D), and an ELISA showed that HGF protein expression in the livers of rats that received unmodified ADSCs was lower than in rats that received HGF-overexpressing ADSCs; however, HGF protein expression was lower in rats in the lenti-shHGF + ADSC group (Fig. 2E).

### HGF Gene-Modified ADSCs Inhibit Radiation-Induced Apoptosis of Hepatocytes

**Figure 3:**
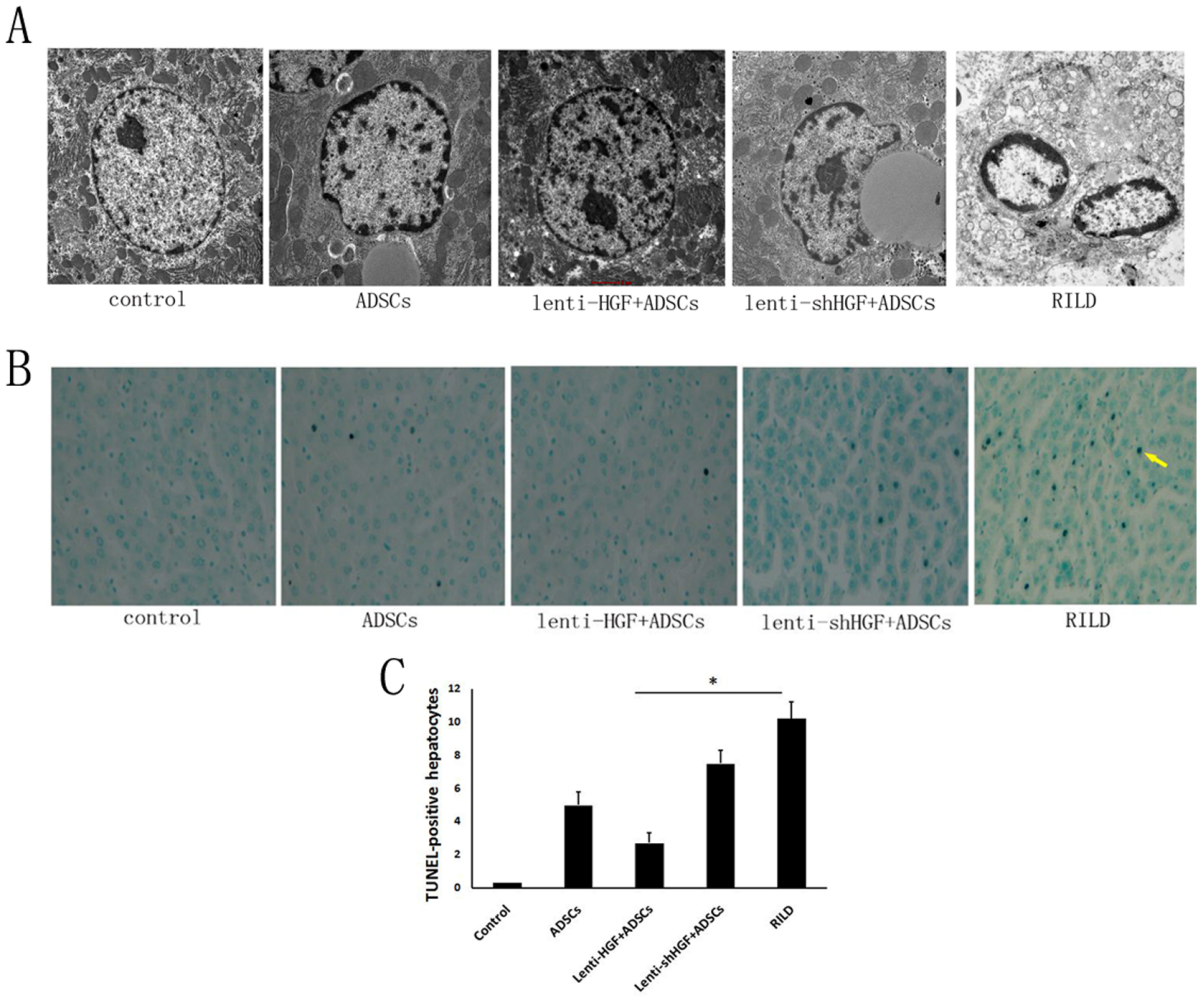
Evaluation of hepatocyte apoptosis 2 days after irradiation. (A) Images of hepatocytes from an electron microscope (×8200). (B) TUNEL staining of liver specimens from all five groups (×400). (C) Quantification of cells that were positive for TUNEL staining: five random fields (200×) were selected from within each liver section, and TUNEL-positive cells were counted. Data are expressed as the mean ± SD. *p<0.05.

Two days after irradiation, five rats from each group were sacrificed to evaluate hepatocyte apoptosis. Electron microscopy was used to observe apoptotic cell morphology, and TUNEL staining was performed to quantify the apoptosis. Electron microscopic scans of liver specimens from rats from the lenti-HGF + ADSC group and from the ADSC group showed significant improvements in nuclear morphology (Fig. 3A). Only slight chromatin condensation was observed in rats from the ADSCs group. The transplantation of ADSCs that overexpressed HGF nearly reversed the apoptotic damage induced by the irradiation. In contrast, in rats from the HGF-knock out group and rats from the RILD group, the liver cells exhibited swollen endoplasmic reticula and Golgi apparatus. TUNEL staining (Fig. 3B) showed that hepatocyte apoptosis was significantly decreased in rats from the ADSC group compared with rats from the RILD group (5.0±0.8 vs. 10.2±1.7, *p*<0.05) (Fig. 3C). The number of TUNEL-positive cells was even lower in rats from the lenti-HGF + ADSC group than rats from the ADSC group (2.7±0.6 vs. 5.0±0.8, *p*<0.05). The number of TUNEL-positive cells in rats from the lenti-shHGF + ADSC group was also decreased compared with rats from the RILD group (7.5±0.8 vs. 10.2±1.7, *p*<0.05), but the anti-apoptotic effect was not equal to that observed in the ADSC group (7.5±0.8 vs. 5.0±0.8, *p*<0.05) (Fig. 3C).

These data indicated that HGF mediates the anti-apoptotic effects of ADSCs in this rat model of radiation-induced liver damage.

### HGF Gene-Modified ADSCs Ameliorate Radiation-Induced Liver Fibrosis

**Figure 4:**
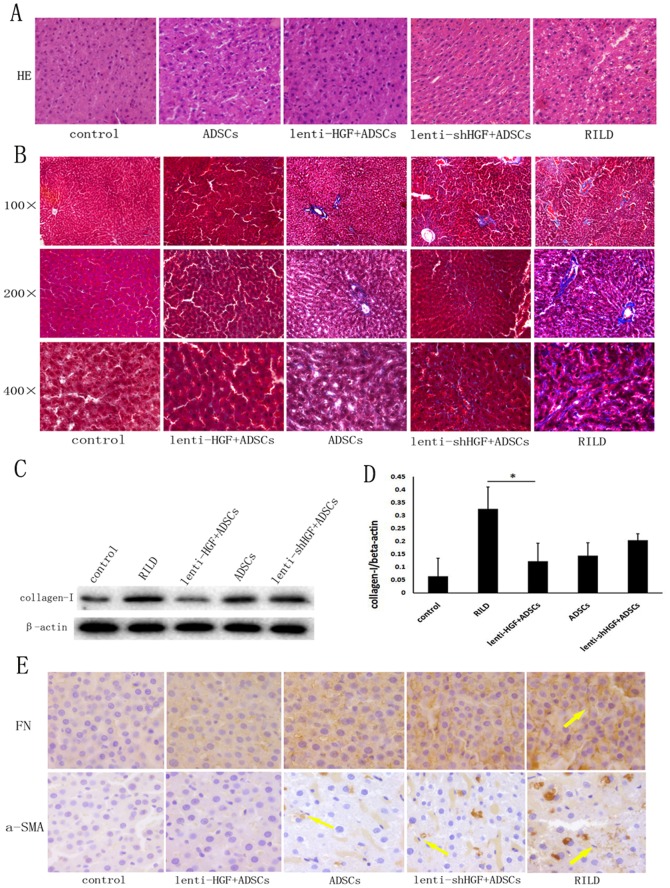
Histological examination of the liver 60 days after irradiation. (A) Histopathology of liver sections stained with hematoxylin-eosin (HE) (400×). (B) Masson's trichome stain of liver sections. (C) Expression of collagen-I in rat livers detected by western blot. (D) Quantification of collagen-I protein expression detected by western blot, *p<0.05. (E) Histopathology of liver sections immunostained with antibodies to fibronectin (FN) and α-SMA (400×). Arrows indicated positive stain of fibronectin (in the images of FN) and α-SMA (in the images of α-SMA).

Because radiation induces fibrosis in the liver, we performed fibrosis detection assays to determine whether ADSCs that overexpress HGF can mitigate fibrotic changes induced by the radiation. Histological examination of liver sections from RILD animals showed liver congestion and hepatocellular ballooning. All three groups that were treated with ADSCs showed amelioration in hepatocellular ballooning. HGF gene-modified ADSCs even abrogated the vacuolar degeneration caused by the irradiation (Fig. 4A). To demonstrate the extracellular collagen deposits in the liver tissue, we performed Masson's trichome staining. Our results demonstrate that radiation can induce collagen deposition in the portal and perisinusoidal regions. However, liver cirrhosis, which is characterized by the formation of a pseudolobule, was not observed 60 days after irradiation. Compared to that of the RILD group, the collagen deposit in the ADSC group was reduced. Almost no collagen accumulation was observed in the lenti-HGF + ADSCs groups (Fig. 4B). Western blot was used to quantify the protein expression (Fig. 4C). A significant up-regulation of collagen-I protein expression was observed in rats from the RILD group after irradiation (0.33±0.09 vs. 0.06±0.07, *p*<0.05), but this protein was significantly reduced in rats from the lenti-HGF + ADSC group (0.12±0.07 vs. 0.33±0.09, *p*<0.05) (Fig. 4D). The activation of hepatic stellate cells is a major event that is associated with the process of liver fibrosis, and α-SMA is up-regulated during the activation of these cells. As revealed in Fig. 4E, there were few a-SMA positive regions in the control group. In contrast, considerable a-SMA-positive regions can be seen around the perisinusoidal areas in the RILD group, whereas the treatment of HGF over-expressing ADSCs attenuated the expression of a-SMA and fibronectin compared to that of the RILD group. Immunohistochemical staining for fibronectin revealed that radiation-induced liver fibrosis was mitigated by the administration of ADSCs and lenti-HGF + ADSCs (Fig. 4E).

### HGF Gene-Modified ADSCs Promote Hepatocyte Regeneration after Irradiation

**Figure 5:**
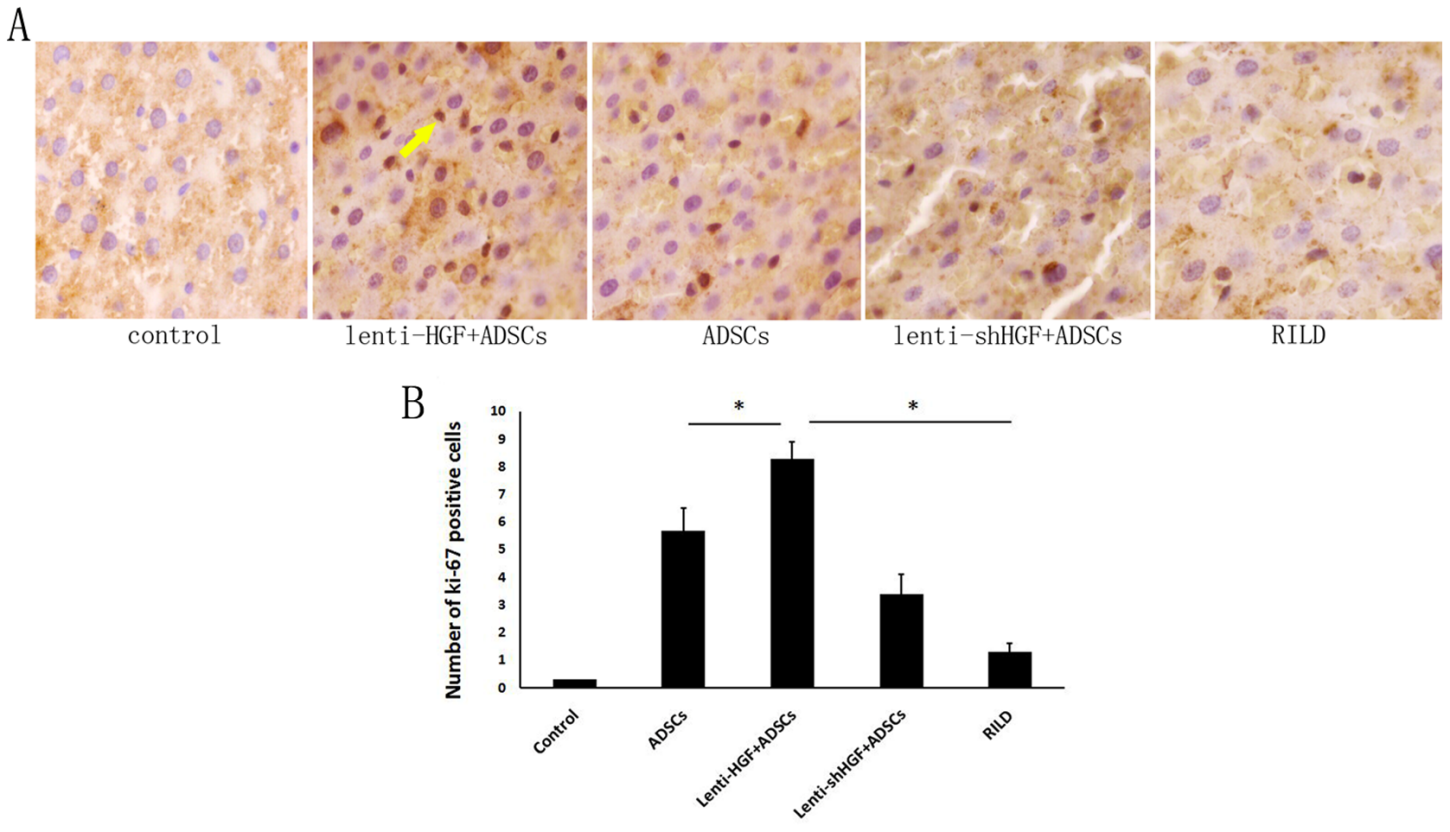
Assessment of hepatocyte proliferation. (A) Histological analysis of liver sections stained with a Ki-67 antibody (400×). Arrow indicates hepatocytes that are positive for Ki-67 staining. (B) Quantification of Ki-67-positive cells. Five random fields (400×) were selected from within each liver section, and the number of Ki-67-positive cells was counted. Data are expressed as the mean ± SD. *p<0.05.

Radiation initiates cellular apoptosis and eventually results in parenchymal cell loss and fibrosis. The reduction in the cell population was histologically observed after irradiation. Ki-67 is a nuclear antigen that is used to detect proliferating cells (Fig. 5A). Five random fields (200×) were selected from within each liver section, and Ki-67-positive cells were counted. Ki-67-positive cells were seldom observed in sections from the RILD group (1.3±0.3) or in sections from the control group (0.3±0.02). On the contrary, the regeneration of hepatocytes labeled with the Ki-67 antibody was shown in all three groups that received cell therapy. The number of Ki-67-positive cells was significantly increased in rats from the ADSC group compared to rats from the RILD group (5.7±0.8 vs. 1.3±0.3, p<0.05); however, the number of Ki-67-positive cells in rats from the ADSC group was lower than in rats from the lenti-HGF + ADSC group (5.7±0.8 vs. 8.3±0.6, p<0.05) but higher than in rats from the lenti-shHGF + ADSC group(5.7±0.8vs. 3.4±0.7, p<0.05) (Fig. 5B).

These results indicated that ADSCs can improve hepatocyte regeneration after irradiation. HGF-overexpressing ADSCs demonstrated an even better efficacy in the regeneration of hepatocytes, and the inhibition of HGF expression substantially weakened the ability of the ADSCs to promote hepatocyte regeneration.

### HGF Gene-Modified ADSCs Improve Liver Function after Irradiation

**Figure 6:**
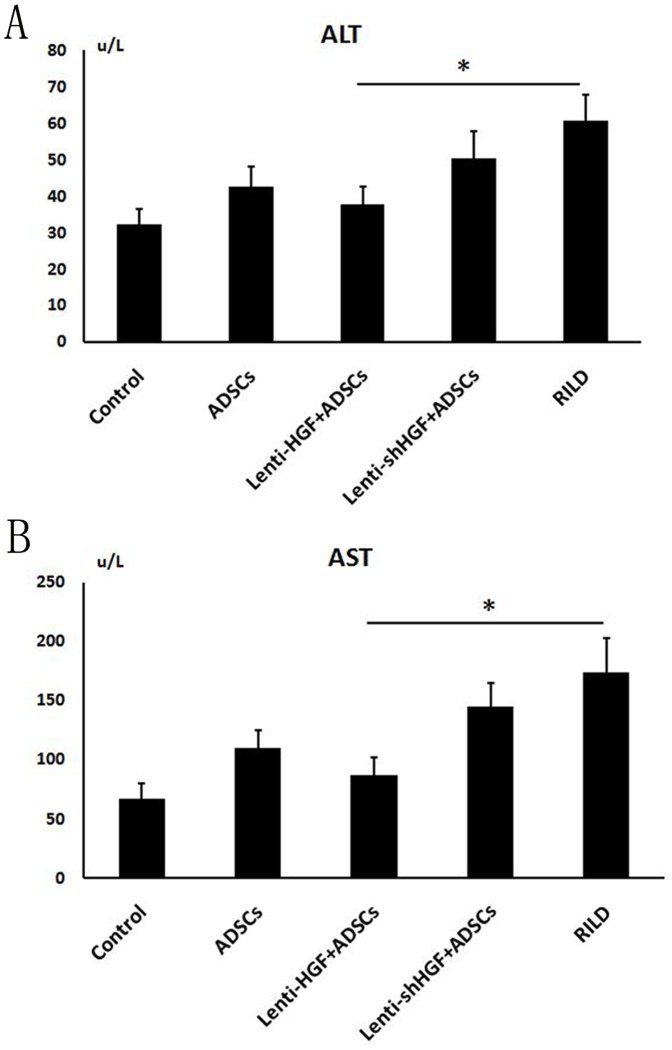
Analysis of liver function index. Serum levels of ALT (A) and AST (B) 2 days after irradiation are shown. *p<0.05.

A biochemical analysis of the liver function index was also performed to assess the protective efficacy of ADSCs against radiation-induced liver damage. The results showed a rise in the levels of ALT from 32.4±4.2 units/L (control) to 60.8±7.2 units/L and a rise in AST from 67.2±12.5 units/L (control) to 174.2±28.4 units/L 2 days post-irradiation. Notably, treatment with HGF-overexpressing ADSCs completely blocked the radiation-induced increase in the enzymes ALT (37.8±4.8 units/L vs.60.8±7.2; Fig. 6A) and AST (87.4±14.4 vs. 174.2±28.4; Fig. 6B). Treatment with ADSCs partially decreased the levels of ALT (42.7±5.3 units/L) and AST (109.7±15.6 units/L) compared with the RILD group, which was not treated with ADSCs (*p*<0.05). The levels of ALT (50.4±7.4 units/L) and AST (145.4±19.5 units/L) were also lower in rats from the lenti-shHGF + ADSC group compared with rats from the RILD group but were higher than the levels in rats from the ADSC group. These data suggested that the administration of lenti-HGF + ADSC prevented radiation-induced liver injury.

## Discussion

In this study, we investigated the therapeutic potential of HGF-overexpressing ADSCs on RILD. It was demonstrated that HGF-overexpressing ADSCs are better than unmodified ADSCs for mediation of a significant therapeutic effect in the treatment of radiation-induced rat liver damage. This mediation occurred through anti-apoptotic and anti-fibrotic mechanisms as well as through the promotion of hepatocyte regeneration. Inhibition of HGF expression compromised the effects of ADSCs, which indicated that HGF, at least in part, plays a crucial role in the therapeutic effect exerted by ADSCs on RILD.

RILD, which is pathologically characterized by veno-occlusive disease (VOD), manifests after exposure of the liver to radiation. Dosages that varied from 260 cGy to 60 Gy were used to generate RILD in previous studies. In this study, we compared 30 Gy and 60 Gy to observe the resulting liver damage and chose a dose of 60 Gy to establish liver damage in our model. The liver exhibits hepatic sinus congestion, hepatocyte necrosis, apoptosis and loss of parenchymal cells in the early stages after irradiation. Then, fibrous tissue accumulates and eventually develops into irreversible liver fibrosis. Therefore, the prevention of apoptosis and fibrosis is key to alleviating RILD.

Mesenchymal stem cells originate from mesoblastic stem cells. They are able to self-renew and differentiate into various cell types, such as osteocytes, adipocytes, chondrocytes, and myocytes, in different inducing environments. MSCs are the main cell type involved in tissue repair. When a tissue injury occurs, bone marrow-derived stem cells (BMSCs) migrate to the location of the damage where they participate in tissue repair [30]. Based on increasing experimental evidence, MSCs are now accepted as an ideal cell line for tissue repair. Of the various mesenchymal stem cell lines, ADSCs have been considered one of the most viable potential therapies for tissue injury due to their abundance and easy access [12]. ADSCs resemble BMSCs in both their self-renewal and differentiation potential. Research has revealed that ADSCs express the same surface markers as BMSCs [31]. Similarly, in this study, ADSCs were shown to express CD90 but did not express CD31, CD34, or CD45, which was consistent with previous studies.

Many studies have demonstrated that MSCs have considerable effects on liver injury [10], [13], [32]. A number of different mechanisms contribute to the therapeutic effects that are exerted by MSCs, which can differentiate into functional hepatic cells and also produce a series of growth factors and cytokines that can ameliorate liver injury. Aurich et al. demonstrated that both MSC-derived hepatocytes and MSCs can be engrafted into the recipient liver and express HepPar1 and albumin, which are typical features of differentiated human hepatocytes [33]. Recent studies have reported that MSCs can produce some antiapoptotic cytokines, such as stromal-cell-derived factor-1 and vascular endothelial growth factor, which efficiently reduce the apoptosis of recipient cells via the stromal cell-derived factor-1/CX chemokine receptor-4 axis [34], [35], [36]. Furthermore, MSCs can secrete several cytokines, such as interleukin 1 receptor alpha (IL-1Ralpha), IL-6, IL-8, granulocyte colony-stimulating factor (G-CSF), nerve growth factor, and hepatocyte growth factor, and these cytokines improved hepatocyte function, as indicated by changes in the levels of biochemical parameters upon MSC transplantation [37]. Moreover, MSCs were more resistant to reactive oxygen species in vitro, reduced oxidative stress in recipient mice, and accelerated the repopulation of hepatocytes after liver damage, suggesting a possible role for paracrine effects [38]. Additionally, clinical trials have demonstrated that MSC injection can be used for the treatment of liver diseases with satisfactory therapeutic effect and tolerability [39], [40]. However, there have been few studies that have focused on the therapeutic potential of ADSCs for RILD.

ADSCs can help to inhibit apoptosis through the regulation of proteins involved in apoptosis. Liu [41] found that ADSCs prevented cell apoptosis via an increase in Bcl-2 expression, which exerted anti-apoptotic effects in a rat cerebral ischemia reperfusion injury model; additionally, ADSCs ameliorate inflammatory reactions, reduce apoptosis and mitigate renal ischemia reperfusion injury through the up-regulation of TNF-α and Bcl-2 [42]. Through the secretion of many protective cytokines, ADSCs treated with low levels of oxygen can also decrease the number of apoptotic myocardial cells, which may be associated with activation of the JNK signaling pathway [43]. In our study, ADSCs bestowed anti-apoptotic effects in a rat model of RILD, which has not been investigated in previous studies.

MSCs potentially reverse liver fibrosis. MSCs have the potential to reverse the fibrotic process by inhibiting collagen deposition and transforming growth factor-β1, Smad2, and α-SMA production. Inhibiting the transforming growth factor-β1-Smad signaling pathway may account for the anti-fibrosis effect of MSCs [44]. The molecular mechanisms underlying the anti-fibrotic properties of MSCs can mainly lie in the high expression levels of matrix metalloproteinases (MMPs), especially MMP-9, which may directly degrade the extracellular matrix and lead to hepatic stellate cell apoptosis [45]. Moreover, Pan et al. reported that BM-MSCs were able to attenuate liver fibrosis via the direct suppression of hepatic stellate cell activation through the inhibition of delta-like 1 protein, a member of the EGF-like family of homeotic proteins, in a CCL4-induced liver fibrosis animal model [46]. However, few previous studies have focused on the effect of ADSCs for the treatment of fibrosis caused by irradiation. In the liver, late stages of radiation-induced injury is histologically characterized by both paracentral and periportal fibrosis. The activation of hepatic stellate cells and collagen deposition are usually involved in the process of liver fibrosis. In this study, we tested for the expression of α-SMA, which is associated with the activation of hepatic stellate cells. We also observed the expression of collagen-I and fibronectin in the liver. ADSCs were able to significantly mitigate radiation-induced liver fibrosis by inhibiting the α-SMA, collagen-I and fibronectin expression. The appearance of proliferating bile ductular structures is frequently observed in various chronic liver diseases associated, including chronic hepatitis and cirrhosis. This process is called the “ductular reaction” [47], [48]. In chronic liver disease, there is association between ductular reaction (DR) and fibrosis [49]. In our study, however, we did not observe observed any positive stain that represents ductular reaction in this model. Meanwhile, most studies did not observe the manifestation of ductular reaction in radiation induced liver injury model, too. This result may due to the differences between different types of liver fibrosis. And further studies are needed to determine whether this model was associated with expansion of the canals of Hering by measuring the ductular reaction, and whether this was changed with therapy.

As for the therapeutic mechanism of MSCs, previous studies tended to consider differentiation as the primary way that these cells promote tissue repair. However, with increasingly deeper insight, it has been accepted that differentiation and paracrine mechanisms cooperate in the promotion of tissue repair; paracrine mechanisms are thought to be even more crucial [30]. In a model of acute renal failure, BMSCs were determined to have anti-inflammatory properties, and they significantly improved the renal function of recipients through paracrine mechanisms [50]. Through the secretion of cytokines, BMSCs play an important role in the migration of endothelial and epithelial cells and in angiogenesis in the promotion of skin lesions [51]. In accordance with these results, Cai [25] found that the ability of ADSCs to promote angiogenesis was impaired after the inhibition of HGF secretion, which implies that paracrine effects may be one of the key mechanisms involved in tissue repair by ADSCs.

Currently, it is believed that MSCs secrete HGF, vascular endothelial growth factor, epidermal growth factor, transforming growth factor (TGF)-β1, insulin-like growth factor -1, MMP family proteins, and many other cytokines that are used to repair injured tissue, among which HGF is closely related to the repair of liver injuries. The expression of HGF would reactively increase after liver injury to promote cell survival and regeneration. An in vivo study has shown that HGF protects liver cells from injury via the promotion of hepatocyte proliferation and through the enhancement of hepatocyte resistance to liver toxicity. Moreover, HGF also inhibits liver fibrosis and the apoptosis of hepatocytes. HGF, by binding to its receptor c-met, activates the PI3K pathway, which stimulates the downstream Akt pathway to inhibit cell apoptosis. HGF also inhibits the activation of hepatic stellate cells and collagen deposition in the liver, which reduces the amount of extracellular matrix and improves liver fibrosis by down-regulating the expression of TGF-β1. As expected, in this study, we found that HGF magnified the effect of ADSCs with respect to anti-apoptotic and anti-fibrotic properties and the promotion of hepatocyte regeneration. Some studies have shown that HGF not only is beneficial for liver injuries but can also effectively improve irradiation-induced tissue damage. Through the inhibition of hepatocellular apoptosis and the down-regulation of TGF-β1 expression, HGF can significantly improve radiation-induced liver damage [52]. Additionally, research has shown that HGF cannot only protect skin cells and endothelial cells from irradiation but can also significantly enhance the generation of myocardial cell proteins after gamma ray irradiation [53], [54]. HGF also plays a crucial role in the mobilization, migration and homing of MSCs [55]. Therefore, in this study, we enhanced the expression of HGF in ADSCs using a lentivirus and thus hoped to achieve better therapeutic efficacy. HGF-overexpressing ADSCs displayed a better therapeutic efficacy in the improvement of angiogenesis and heart function compared with unmodified ADSCs when applied to a model of myocardial ischemia [24]. The superiority of HGF-overexpressing ADSCs was also demonstrated in a rat model of bladder outlet obstruction [56]. In this study, compared with unmodified ADSCs, HGF-overexpressing ADSCs were more effective in the treatment of radiation-induced liver damage. Nevertheless, ADSCs can secrete other cytokines with multiple biological functions. Whether their expression or inhibition can affect the efficacy of ADSCs on RILD remains unknown, and as such, further studies are needed.

In this study, we found that ADSCs demonstrate significant therapeutic effects in the treatment of RILD possibly through the prevention of apoptosis and liver fibrosis and through the promotion of hepatocyte regeneration. HGF-overexpressing ADSCs demonstrated even better therapeutic efficacy on RILD. HGF demonstrated important effects in the treatment of irradiation-induced liver damage and provides a novel method for the treatment of RILD in clinical practice.
